# Regulation of microRNA expression in the neuronal stem cell niches during aging of the short-lived annual fish *Nothobranchius furzeri*

**DOI:** 10.3389/fncel.2014.00051

**Published:** 2014-02-21

**Authors:** Eva Terzibasi Tozzini, Aurora Savino, Roberto Ripa, Giorgia Battistoni, Mario Baumgart, Alessandro Cellerino

**Affiliations:** ^1^Laboratorio di Biologia, Scuola Normale Superiore Pisa, Italy; ^2^Fritz Lipmann Institute for Age Research, Leibniz Institute Jena, Germany

**Keywords:** microRNA regulation, *Nothobranchius furzeri*, adult neurogenesis, aging, neuronal stem cells, maturation, *in situ* hybridization

## Abstract

In the last decade, our group has intensively studied the annual fish *Nothobranchius furzeri* as a new experimental model in Biology specifically applied to aging research. We previously studied adult neuronal stem cells of *N. furzeri in vivo* and we demonstrated an age-dependent decay in adult neurogenesis. More recently we identified and quantified the expression of miRNAs in the brain of* N. furzeri* and we detected 165 conserved miRNAs and found that brain aging in this fish is associated with coherent up-regulation of well-known tumor suppressor miRNAs, as well as down-regulation of well-known onco miRNAs~– In the present work we characterized the expression of miR-15a, miR-20a, and microRNA cluster 17–92 in the principal neurogenic niches of the brain of young and old subjects of *N. furzeri*, by using *in situ* hybridization techniques, together with proliferating-cell nuclear antigen immuno-staining for a simultaneous visualization of the neuronal progenitors. We found that: (1) the expression of miR-15a is higher in the brain of old subjects and concentrates mainly in the principal neurogenic niches of telencephalon and optic tectum, (2) the expression of miR-20a is higher in the brain of young subjects, but more widespread to the areas surrounding the neurogenic niches, (3) finally, the expression of the microRNA cluster 17–92 is higher in the brain of young subjects, concentrated mainly in the principal neurogenic niches of telencephalon and cerebellum, and with reduced intensity in the optic tectum. Taken together, our data show that these microRNAs, originally identified in whole-brain analysis, are specifically regulated in the stem cell niche during aging.

## INTRODUCTION

MicroRNAs (miRNAs) are abundant non-coding RNAs around 20–22 nucleotides in length, which are emerging as important key players in the regulation of gene expression. miRNAs are transcribed by RNA Polymerase II (i.e., the same Polymerase which transcribes protein-coding RNAs) as long transcripts called primary transcripts and undergo a complex processing before being included in a ribonucleic complex. Several miRNAs can be grouped in a genomic cluster and co-transcribed, and may be hosted within an intron of a protein-coding gene. MiRNAs bind, due to sequence complementarity, to specific sites in the 3′ untranslated region of their target mRNAs, thereby silencing expression of the gene product via translational repression and/or mRNA degradation. Indeed, they represent a new level of gene regulation acting at the post-transcriptional level. Up to now, several thousands of miRNAs have been predicted and identified in animals, plants and viruses (http://www.mirbase.org).

A feature of miRNAs is their combinatorial regulation: a given miRNA can target a multitude of different mRNAs and a given target might similarly be targeted by multiple miRNAs; for this reason, they frequently represent the central nodes of several regulatory networks and may act as rheostat to provide stability and fine-tuning to gene expression networks (Osella et al., 2011; Siciliano et al., 2013). Moreover, they are promising candidates for functional studies by genome-wide transcriptional analysis, thanks to some specific features: (i) miRNAs are highly conserved in vertebrates (cases of 100% identity between fish and mammals are not uncommon) and are thought to be an evolutionarily ancient component of genetic regulation; (ii) in a single tissue, relatively few miRNAs are expressed (hundreds vs. tenths of thousands mRNAs); (iii) they represent in their context the biologically active molecule, since they directly bind and control the target mRNAs: measurements of miRNA concentrations allow a more direct inference of a biological function.

In the last decade, our group has intensively studied the annual fish *Nothobranchius furzeri* as a new experimental model in Biology. This fish inhabits ephemeral pools in semi-arid *bushveld* of Southern Mozambique characterized by scarce and erratic precipitations and have adapted to the seasonal drying of their environment by producing desiccation-resistant eggs which can remain dormant in the dry mud for one and maybe more years by entering into diapause (Genade et al., 2005). Due to very short duration of the rain season, the natural lifespan of these animals is limited to a few months (Terzibasi Tozzini et al., 2013). They represent the vertebrate species with the shortest captive lifespan and also the fastest maturation (Genade et al., 2005; Blazek et al., 2013). In addition, they express a series of conserved aging markers and are amenable to genetic manipulations, making them an attractive model system for aging research (Valenzano et al., 2006; Hartmann et al., 2009, 2011; Terzibasi et al., 2009; Di Cicco et al., 2011; Valenzano et al., 2011; Hartmann and Englert, 2012; Allard et al., 2013). Fish brains are characterized by a very active adult neurogenesis with stem cell niches distributed along the entire rostro–caudal extent of the ventricular surface (Zupanc and Horschke, 1995; Adolf et al., 2006; Grandel et al., 2006; Kuroyanagi et al., 2010). Adult neurogenesis in mammals is known to decrease dramatically with age (Kuhn et al., 1996; Pekcec et al., 2008; Ben Abdallah et al., 2010; Knoth et al., 2010). Adult neurogenesis in mammals is restricted to two neurogenic niches in the telencephalon (TEL). Adult neurogenesis in teleosts, on the other hand, is widespread along the entire rostro–caudal axis and it is therefore unclear whether the same age-dependent decay is observed as in mammals. We therefore studied adult neuronal stem cells of *N. furzeri in vivo* and demonstrated an age-dependent decay in adult neurogenesis in terms both of incorporation of nucleotide analogs and expression of specific markers. In addition, RNA-seq experiments revealed age-dependent down-regulation of cell cycle genes during aging of *N. furzeri* brain (Petzold et al., 2013). We also observed a dramatic up-regulation of GFAP protein in the radial (neurogenic) glia of aged *N. furzeri* brains. All these data indicate a drastic reduction of neuronal stem cell activity during *N. furzeri* aging (Terzibasi Tozzini et al., 2012).

In the present paper, we use *N. furzeri* to model age-dependent decay of neurogenesis. We specifically analyzed the telencephalic neurogeneic niches that share the same embryonic origin with the mammalian adult niches (Adolf et al., 2006) and in particular the ventral niche that is homologous to the subventricular (subpallial) zone and the dorsal region that is homologous to pallial zone. For comparisons, we also analyzed the germinal zone of the optic tectum (OT), that is specific to teleosts and its stem cells do not show a glial phenotype (Terzibasi Tozzini et al., 2012). This would allow us to differentiate between conserved expression patterns in the telencephalic niches from possible teleost-specific expression pattern detectable only in the OT.

We previously used small RNA sequencing to identify and quantify expression of miRNAs in the brain of *N. furzeri* using miRBase as reference. We could detect 165 conserved miRNAs and found that brain aging in *N. furzeri* is associated with coherent up-regulation of well-known tumor suppressor miRNAs (such as miR-15a) that show positive interactions with TP53 and negative interactions with MYC, while the opposite is true for down-regulated miRNAs, such as miR-20a and other miRNAs belonging to the miRNA cluster 17–92 (Baumgart et al., 2012). Further, several of these miRNAs are regulated in the primate brain as well (Somel et al., 2010). This regulation is probably linked to the age-dependent reduction in adult neurogenesis observed in *Nothobranchius* species, as it would be suggested also by down-regulation of miR-9, a miRNA enriched in adult neuronal precursors (Terzibasi Tozzini et al., 2012).

The prototypic miRNA let-7 was originally shown to regulate the timing of developmental events in *Caenorhabditis elegans* (Reinhart et al., 2000) and later to regulate the age-dependent loss of regeneration in *C. elegans* (Zou et al., 2013). Studies in Vertebrates have indicated that the timing of miRNAs expression can regulate the timely generation of neurons with different cell-fates (Cremisi, 2013). Aim of the present work was to characterize the expression pattern for some miRNAs which are regulated during aging of *N. furzeri* brain, focusing on miRNAs known to be regulators of the cell cycle, since it has been previously demonstrated (Terzibasi Tozzini et al., 2012) that neuronal stem cells (NSCs) decrease with age. The miRNA cluster 17–92 is the prototypical oncogenic miRNA *locus* and it is up-regulated in a variety of cancers. It codes for six miRNAs: miR-17a, miR-18a, miR-19a, miR-20a, miR-19b, and miR-92a (Mogilyansky and Rigoutsos, 2013) and this organization is conserved in teleost fish (Guo et al., 2013). Its expression is controlled by the prototypical oncogene MYC and it targets known oncosuppressors such as PTEN and CDKN1A and pro-apoptotic genes such as BCL2L11 thereby contributing to oncogenic transformation (Mogilyansky and Rigoutsos, 2013). The miRNA cluster 17–92 is also associated to aging, as it is down-regulated during senescence of human cells both *in vitro* and *in vivo* (Hackl et al., 2010), cardiac aging in mice (van Almen et al., 2011) and aging of *N. furzeri* brain (Baumgart et al., 2012). Recent studies have shown that miR-17–92 cluster also regulates neuronal stem cell expansion and axonal elongation of embryonic neuronal precursors via targeting of PTEN (Bian et al., 2013; Zhang et al., 2013). On the other side, miR-15a is a known oncosuppressor that is regulated by TP53 and targets cell-cycle and anti-apoptotic proteins (Finnerty et al., 2010). Action of miR-15a in the nervous system is unknown, but during normal development of the heart miR-15a induces mitotic cycle exit of postnatal cardiomyocytes (Porrello et al., 2011). Expression of miR-15a increases during aging of *N. furzeri* (Baumgart et al., 2012) and may contribute to reduced activity of neuronal stem cells. We therefore decided to analyze miR-15a and, on the other side, miR-20a and cluster 17–92, for more in-depth investigations and analyzed their expression at the cellular level in the neurogenic niches of young- and old-fish.

## RESULTS

We analyzed the expression of miR-15a, miR-20a and miRNA cluster 17–92 in the brain of young and old subjects of *N. furzeri*, by using *in situ* hybridization techniques, together with proliferating-cell nuclear antigen (PCNA) immuno-staining for a simultaneous visualization of the neurogenetic niches.

### CLONING OF THE GENOMIC microRNA CLUSTER 17–92 From *N. furzeri*

**FIGURE 1:**
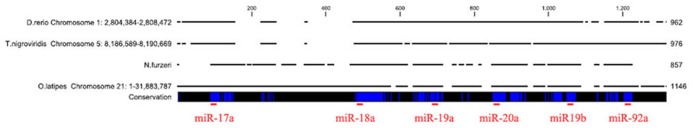
**Alignment of the microRNA cluster 17–92 from the four teleost species.** The genomic regions of *D. rerio*, *T. nigroviridis*, and *O. latipes* were aligned to the fragment amplified in *N. furzeri*. The blue vertical bars indicate identical nucleotides. The localization of the mature miRNA sequences is indicated by a red horizontal bar.

**FIGURE 2:**
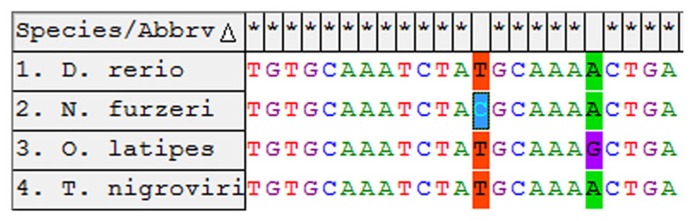
**Alignment of the miR-19a-3p from the four teleost species.** The non-conserved sites are indicated in color.

In order to isolate the miRNA cluster 17–92 from *N. furzeri,* we designed primers covering the sequences of miR-17-5p and miR-92a-3p (**Figure 1**). The primers amplified a fragment of 857 bp. Alignment of this fragment with the corresponding genomic regions of *Oryzias latipes*, *Tetraodon nigrovirids* and *Danio rerio* revealed the expected conservation in the regions of all the pre-miRNA of the cluster. Alignment of the sequences corresponding to the mature miRNAs of the cluster revealed 100% conservation in teleosts of miR-17, miR-18a, miR-20a, miR-19b, and miR-92a. The sequence of miR-19a showed a single position variation both in *N. furzeri *compared with the other three species (13 T > C, **Figure 2**) and in *O. latipes* compared with the other three species (19 A > G, **Figure 2**).

### EXPRESSION OF miR-15a IS HIGHER IN THE BRAIN OF OLD SUBJECTS AND CONCENTRATES MAINLY IN THE PRINCIPAL NEUROGENETIC NICHES OF TEL AND OT

**FIGURE 3:**
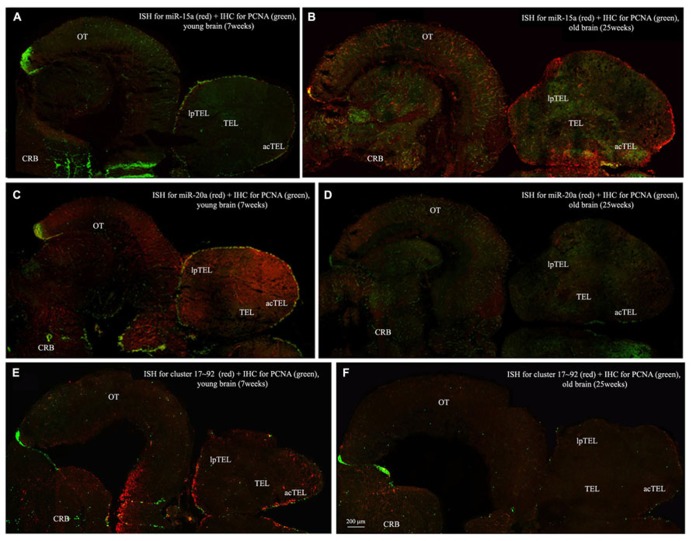
**Overviews of *Nothobranchius furzeri* hemi-brain.** In situ Hybridization (ISH, in red) for miR15 **(A,B)**, miR20 **(C,D)** and Cluster17–92 **(E,F)**, double stained with PCNA (in green) by immunohistochemistry (IHC), in young (**A,C,E**, 7 weeks old) versus old (**B,D,F**, 25 weeks old) subjects. TEL, telencephalon; acTEL, antero-central telencephalon; lpTEL, latero-posterior telencephalon; OT, optic tectum; CRB, cerebellum.)

**FIGURE 4:**
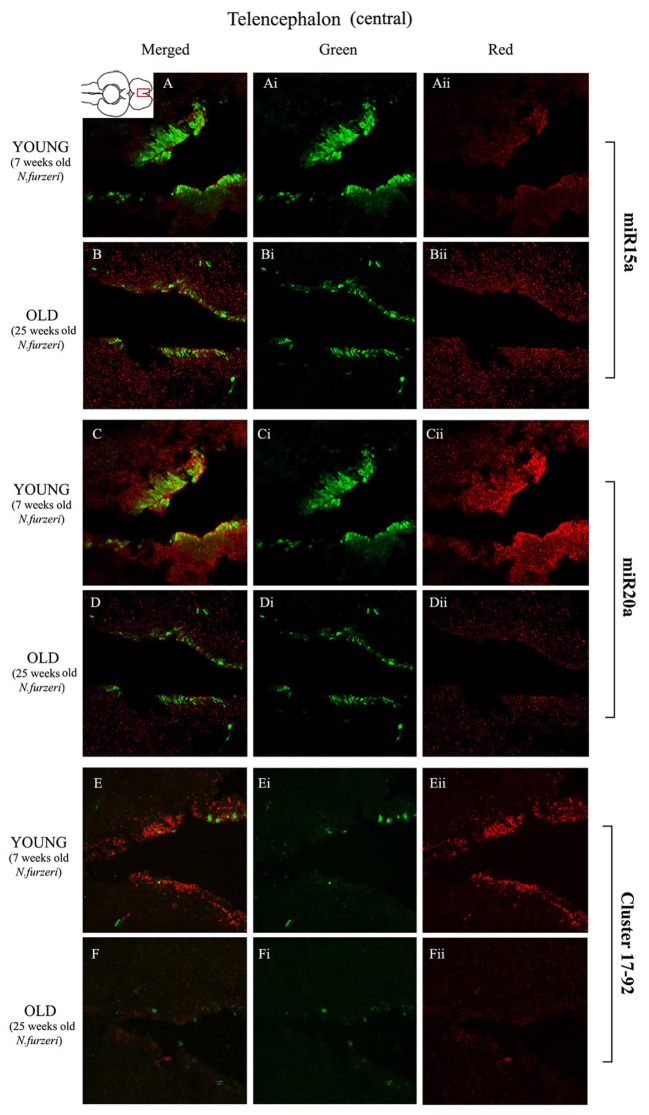
**Magnification of the central region of the telencephalon (cTEL) stained in green for PCNA by IHC, and in red for miR15 (A,B strips), miR20 (**C,D** strips) and Cluster17–92 (**E,F** strips) by ISH.** The upper inset on **A** shows an overview of a horizontal brain section: the location of the cTEL is indicated by the red rectangle. The left column of the panel **(A–F)** shows the merged channels for the double staining; **A,B** refer to ISH for miR15a, respectively, in a young versus an old representative subject. **C,D** refer to ISH for miR20a, respectively, in a young versus an old representative subject. **E,F** refer to ISH for Cluster17–92, respectively, in a young versus an old representative subject. Central (**Ai**–**Fi**) and right (**Aii**–**Fii**) columns show the green and red single channel of the respective image on the left.

**FIGURE 5:**
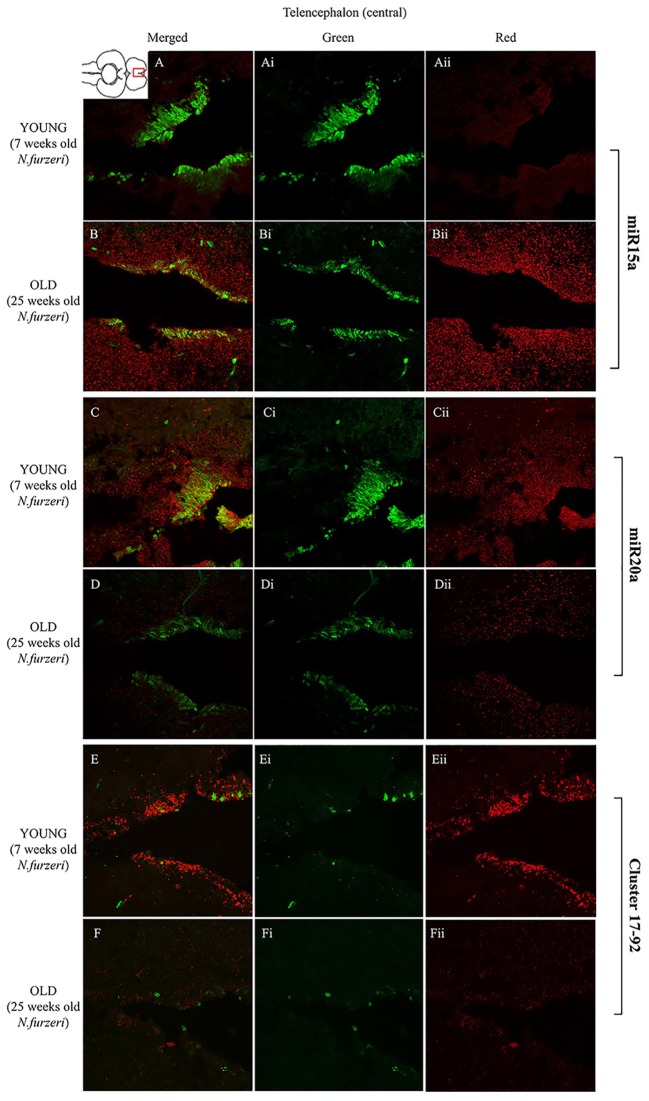
**Magnification of the latero-posterior region of the telencephalon (lpTEL) stained in green for PCNA by IHC, and in red for miR15 (A,B strips), miR20 (**C,D** strips), and Cluster17–92 (**E,F** strips) by ISH.** The lower inset on **Aii** shows an overview of a horizontal brain section: the location of the lpTEL is indicated by the red rectangle. The left column of the panel (**A**–**F**) shows the merged channels for the double staining; **A,B** refer to ISH for miR15a, respectively, in a young versus an old representative subject. **C,D** refer to ISH for miR20a, respectively, in a young versus an old representative subject. **E,F** refer to ISH for Cluster17–92, respectively, in a young versus an old representative subject. Central (**Ai**–**Fi**) and right (**Aii**–**Fii**) columns show the green and red single channel of the respective image on the left.

**FIGURE 6:**
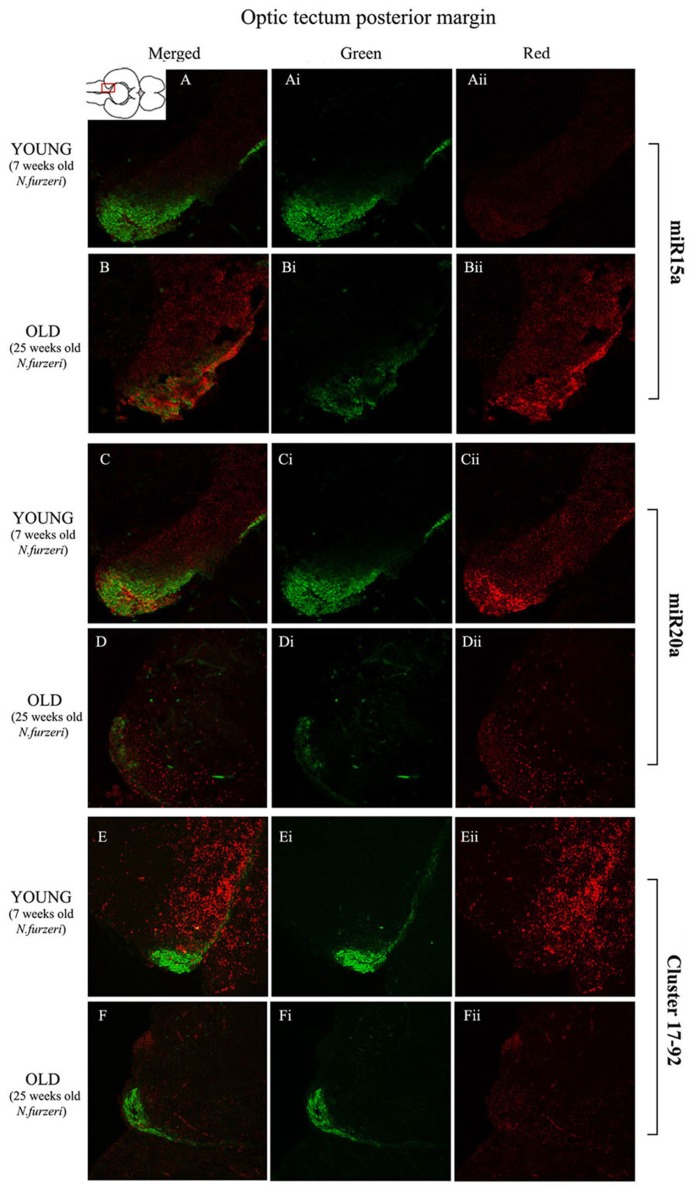
**Magnification of the posterior margin of the optic tectum (pOT) stained in green for PCNA by IHC, and in red for miR15 (A,B strips), miR20 (**C,D** strips) and Cluster17–92 (**E,F** strips) by ISH.** The upper inset on **A** shows an overview of a horizontal brain section: the location of the pOT is indicated by the red rectangle. The left column of the panel (**A**–**F**) shows the merged channels for the double staining; **A,B** refer to ISH for miR15a, respectively, in a young versus an old representative subject. **C,D** refer to ISH for miR20a, respectively, in a young versus an old representative subject. **E,F** refer to ISH for cluster17–92, respectively, in a young versus an old representative subject. Central (**Ai**–**Fi**) and right (**Aii**–**Fii**) columns show the green and red single channel of the respective image on the left.

To visualize expression of miR-15a in *N. furzeri* brain we used a probe for fru-miR-15a whose sequence is identical to that of *N. furzeri.* **Figures 3A,B** show an overview of miR-15a distribution in young- and old-subjects, respectively, in a low-power overview and double labeling. The neurogenic activity is detected by immunohistochemistry for PCNA and visualized as a green fluorescence staining and labeling for the miRNA obtained using and LNA probe is shown as red fluorescence. Three regions of interest are indicated in the hemi-brains horizontal section, and represent areas were some of the most active neurogenetic niches are located (Terzibasi Tozzini et al., 2012): TEL and relative sub-regions (acTEL, antero-central TEL; lpTEL, latero-posterior TEL), OT and Cerebellum (CRB). As expected from previous results (Terzibasi Tozzini et al., 2012), PCNA positive cells (green) are more numerous in all neurogenic areas of the young brains (**Figures 3A,C,E**) as compared to the old ones (**Figures 3B,D,F**). Expression of miR-15a is overlapping with the neurogenic niches but is weaker in the young-subject (**Figure 3A**) as compared to the old-subject (**Figure 3B**). This is in line with the up-regulation of miR-15 detected by qPCR and miRNA-seq (Baumgart et al., 2012). The distribution of miR-15a can be appreciated more in detail in the magnifications of the different regions (**Figures 4–6**): in the TEL, miR-15a expression is primarily concentrated in the acTEL (**Figures 4Bi,ii**) and in the lpTEL (**Figures 5Bi,ii**) neurogenetic niches, and staining is clearly more prominent in the old, as compared to the young subject (**Figures 4Ai,ii** and **5Ai,ii** for acTEL and lpTEL, respectively). In the old OT, miR-15a expression is concentrated in the posterior margin (**Figures 6Bi,ii**, red), co-localizing with the proliferative niche of PCNA positive cells (**Figures 6Bi,ii**, green). Notably, the young subject presents a weaker miR15a expression in the same area.

### EXPRESSION OF miR-20a IS HIGHER IN THE BRAIN OF YOUNG SUBJECTS, BUT MORE WIDESPREAD TO THE AREAS SURROUNDING THE NEUROGENIC NICHES

To visualize expression of miR-20a in *N. furzeri* a LNA probe designed against dre-miR-20a was used. An overview of miR-20 expression into young and old brain is shown in **Figures 3C,D**, respectively, and its presence is clearly higher in the young tissue with respect to the old one, in line with previous results of qPCR and miRNA-seq (Baumgart et al., 2012). Unlike miR-15a, which results highly concentrated into the neurogenic niches, miR-20a shows a more widespread distribution through acTEL, CRB, and OT regions of young subjects. MiR-20a expression can be appreciated more in detail in the magnification panels (**Figures 4–6**): in the acTEL region of the young brain (**Figures 4Ci,ii**) red fluorescence indicates a strong expression of miR-20a into the centro-ventricular neurogenetic niche (evidenced by the green fluorescence of PCNA positive cells) and, at a lower level, in the surrounding areas. Its expression is radically reduced in the same regions of the old brain (**Figures 4Di,ii**). A similar situation can be observed for the lpTEL region (**Figures 5C,D** rows) and the OT (**Figures 6C,D** rows), where the young tissue shows in both cases a stronger and more diffuse miR-20a expression (**Figures 5** and **6C** rows), compared to the old one (**Figures 5** and **6D** rows).

### EXPRESSION OF THE microRNA CLUSTER 17–92 IS HIGHER IN THE BRAIN OF YOUNG SUBJECTS, CONCENTRATED MAINLY IN THE PRINCIPAL NEUROGENIC NICHES OF TELENCEPHALON AND CEREBELLUM, AND WITH REDUCED INTENSITY IN THE OPTIC TECTUM

Finally, we performed an ISH in young- versus old-brains to evaluate the expression of the primary transcript (pri-miRNA) for the miRNA cluster 17–92 during aging. The overview of its expression in young and old hemi-brains is represented in **Figures 3E,F**, respectively. Similarly to miR-20a, the pri-miRNA is more expressed in the young brain as compared to the old one, but in this case its distribution results mainly concentrated into the neurogenic niches of the TEL (better appreciated in the magnifications of the acTEL and lpTEL of **Figures 4** and **5**, respectively, **E** and **F** rows) and CRB (magnifications not shown). Compared to these regions, the young OT is characterized by a weaker, but still present, expression of miRNA cluster 17–92 (magnifications of **Figures 6E,F** rows) partially extended to the region adjacent to the proliferative niche of the posterior margin (**Figure 6E**), and expression is undetectable in the old OT.

## DISCUSSION

The action of miRNA was widely investigated in the context of tumor biology. The role of miRNAs on the context of brain aging and neurogenesis until recently concentrated on miR-9 and miR-124 (Leucht et al., 2008; Cheng et al., 2009; Maiorano and Mallamaci, 2010; Coolen et al., 2012). Here, we used in situ hybridization to localize the cells expressing one prototypical oncogenic miRNA cluster (miR-17–92) and one prototypical oncosuppressor miRNA (miR-15a) in the brain of *N. furzeri* during aging. Both miRNAs were detected preferentially in the neurogenic niches and the sites of expression did not change during aging. However, at a qualitative level, opposing temporal patterns were apparent: during aging, the intensity of labeling for miR-17–92 primary transcripts and miR-20a decreases while the intensity of labeling for miR-15a increases. This is consistent with previous quantitative analysis of miRNA-seq and qPCR of whole brain extracts showing that, during aging, the expression of the miR-17–92 miRNA cluster decreases and the expression of miR-15a increases (Baumgart et al., 2012). The brain of *N. furzeri* contains widespread neuronal stem cells and is characterized by a drastic age-dependent reduction of neurogenesis (Terzibasi Tozzini et al., 2012). It is therefore likely that regulation of these miRNAs in the stem cell niche generates a signal that is detected by quantitative techniques in whole-brain extracts.

The miRNA cluster 17–92 is down-regulated during aging in a variety of models (Hackl et al., 2010; van Almen et al., 2011; Baumgart et al., 2012), but no data are present as to the site of expression of miRNA cluster 17–92 in the brain. We could specifically show that this gene is expressed in the neurogenic nice but also cells surrounding it. This indicates that miRNA cluster 17–92 is important for adult neuronal stem cell function, but remains activates also in newborn neurons. Indeed recent studies have shown that miR-17–92 cluster regulates neuronal stem cell expansion and axonal elongation of embryonic neuronal precursors via targeting of PTEN (Bian et al., 2013; Zhang et al., 2013). In young animals, the expression of miR-20a (one of the members of the cluster) is more widespread that the expression of the primary transcript. This is expected, since primary transcripts have very short half-life while mature miRNAs are thought to be long-lived.

miRNA-15a act as a general negative regulator of the cell cycle (Finnerty et al., 2010) and induces mitotic cycle exit of postnatal cardiomyocytes (Porrello et al., 2011). Therefore, increased expression of miR-15a in the aged neuronal stem cells is consistent with reduced cell cycle activity.

Our data suggest that miR-17–92 cluster and miR-15a play opposing roles in the regulation of stem cell activity and that their age-dependent imbalance is part of the mechanisms responsible for age-dependent reduction of adult neurogenesis.

## MATERIALS AND METHODS

### FISH BREEDING AND HOUSING CONDITIONS

All experiments were performed on group-house *N. furzeri* of the MZM-04/10 strain. The protocols of fish maintenance were carried out in accordance with all animal use practices established by the Italian Ministry of Health (Number 96/2003a).

Eggs were maintained on wet peat moss at room temperature in sealed Petri dishes. When embryos had developed, eggs were hatched by flushing the peat with tap water at 16–18°C. Embryos were scooped with a cut plastic pipette and transferred to a clean vessel. Fry were fed with newly hatched *Artemia nauplii *for the first 2 weeks and then weaned with finely chopped *Chironomus* larvae. Starting at the fourth week of life, fish were moved to 40-l tanks at a maximum density of 20 fish per tank equipped with air-driven sponge filters. The aquarium room’s temperature was set at a constant 26°C. Twice a week the bottom of the tanks was siphoned and 50% of the water was exchanged with tempered tap water.

### TISSUE COLLECTION AND PREPARATION

Fish were euthanized with MS-222 and cooled on crushed ice for 5 min before dissection. Whole brains from young (7 weeks) and old (25 weeks) animals were dissected and fixed by immersion in 4% paraformaldehyde/0.1 M phosphate buffer (pH 7.4), and then cryoprotected with a two-step immersion at 20% and then 30% sucrose solution for at least 12 h each. Finally the tissues were embedded at -20°C in Neg50 crio-embedding medium (Thermo Scientific); series of 16 μm thick sections were cut with a Leica cryostat and collected on Superfrost plus slides^®^ (Thermo Scientific).

### CLONING OF miR-17–92 Locus of *N. furzeri* AND PROBE PREPARATION

PCR was performed on cDNA using GoTaq polymerase (Promega), 56 degrees for annealing temperature, and 60 s elongation time using the following primers:

Forward: CAAAGTGCTTACAGTGCAGGT

T7-Reverse: GTAATACGACTCACTATAGGG-GGCCGGGACAAGTGCAATACC

0.5 μg of PCR products that contain T7 RNA polymerase promoter at the 3′ ends were used as templates for in vitro transcription. Probes were transcribed using DIG RNA labeling kit (SP6/T7) (Roche), according to the manufacturer’s protocol.

The sequence of the *N. furzeri* miR-17–92 locus was deposited in GeneBank (Accession Number: KF986732). LNA probes for the mature form of fru-miR-15a (MI0003469) and dre-miR-20a (MI0001907) were directly ordered from Exiqon (Denmark)

### *IN SITU* HYBRIDIZATION

We performed *in situ* Hybridization using two different probes: classical RNA probes, to detect transduction products expression of specific genes, and LNA probes, to detect the expression of several mature miRNAs (functional form) of our interest.

All *in situ* Hybridization protocols has been performed on 16 μm thick cryo-sections of fish brain. Slides were dried for 2 h at 37°C, washed in PBS twice for 3 min, and then treated for 8 min with Proteinase K (diluted 1:80000 starting from stocks of 20 mg/mL). After that, slides were washed in Glycine (2 mg/mL in PBT) twice for 5 min, to stop the reaction. Then, sections were fixed with PFA 4% for 20 min at room temperature, and washed in PBT (three times for 3 min). Pre-hybridization were performed covering the slides with 200 μl of hybridization buffer under parafilm coverslips (to avoid evaporation) at hybridization temperature (60°C for classic RNA probes; 37°C for LNA probes) for 30 min. Hybridization was performed covering each slide with a solution of the specific antisense probe, or LNA 3′ DIG labeled exiqon probe diluted in both cases in 200 μl of hybridization buffer to a final concentration of 1 μg/mL. Parafilm coverslips were used and slides incubated at hybridization temperature overnight. Before using them, diluted RNA probes (not LNA) have been denatured for 5 min at 80°C. In order to avoid drying out the slides the whole process has been carried out in wet chamber with PBS.

After hybridization, 2× SSC has been used to remove the coverslip. Slides were first washed in 1× SSC, twice for 20 min, and then in 0.2× SSC twice for 20 min, always at hybridization temperature. A final washing step was done in PBT three times for 5 min at room temperature.

For the probe revelation slides were incubated with blocking solution for 30 min at room temperature and then with Anti-Dig-AP Fab Fragments Ab [1/2000] in blocking solution overnight at 4°C.

Washings in PBT, 3 times for 5 min, and in NMNT, 3 times for 5 min at room temperature, have been conducted before adding Fast Red solution (Roche Tablets; 1 in 2 mL Tris-HCl 0.1 M, pH = 8.2). To avoid the formation of precipitate, Fast Red tablets have been vortexed for 5 min in Tris-HCl and then filtered. Observation has been conducted every 20 min with a Zeiss fluorescence microscope until the signal detection (1–10 h depending on the probe used). The staining has been stopped washing well in PBS (at least 3 times for 5 min) at room temperature.

### HISTOLOGY: PCNA STAINING

After ISH procedure, slides were processed to stain the population of proliferating cells in the neurogenetic niches, following the standard procedures of immunohistochemistry**:** we used****the primary antibody against PCNA commercially available from DAKO (mouse monoclonal, clone PC10, code: M0879) diluted 1:500. Visualization of the primary antibody was performed with the secondary antibody Alexa Fluor^®^488 (goat anti-mouse, Life Technologies, code: A10680) diluted 1:400. The staining has been stopped washing well in PBS (at least 3 times for 5 min) at room temperature. Then slides were closed with a specific mounting (Fluoroshield, Sigma) and analyzed with a confocal microscope (Leica TCS).

## AUTHOR CONTRIBUTIONS

Eva Terzibasi Tozzini designed the study, performed *in situ *hybridization, confocal acquisition and imaging, and wrote the paper. Aurora Savino performed most part of the *in situ *hybridization experiments and part of the confocal acquisition for this reason she should be considered co-first author together with Eva Terzibasi Tozzini. Giorgia Battistoni performed *in situ *hybridization. Roberto Ripa and Mario Baumgart performed the cloning of the standard and LNA probes. Alessandro Cellerino designed and supervised the study and wrote the paper. All authors read and approved the final manuscript.

## Conflict of Interest Statement

The authors declare that the research was conducted in the absence of any commercial or financial relationships that could be construed as a potential conflict of interest.
