# Corrigendum: Regulation of microRNA expression in the neuronal stem cell niches during aging of the short-lived annual fish *Nothobranchius furzeri*

**DOI:** 10.3389/fncel.2018.00227

**Published:** 2018-08-06

**Authors:** Eva Terzibasi Tozzini, Aurora Savino, Roberto Ripa, Giorgia Battistoni, Mario Baumgart, Alessandro Cellerino

**Affiliations:** ^1^Laboratorio di Biologia, Scuola Normale Superiore, Pisa, Italy; ^2^Fritz Lipmann Institute for Age Research, Leibniz Institute, Jena, Germany

**Keywords:** microRNA regulation, *Nothobranchius furzeri*, adult neurogenesis, aging, neuronal stem cells, maturation, *in situ* hybridization

In the original article, there was a mistake in Figure 5 as published.

During assembly of this multi-panel figure, some panels were erroneously duplicated and in particular Panels 5A,C and Panels 5B,D, respectively. The corrected Figure 5 is shown at the end of this article.

The authors apologize for this error and state that this does not change the scientific conclusions of the article in any way.

The original article has been updated.

**Figure 5:**
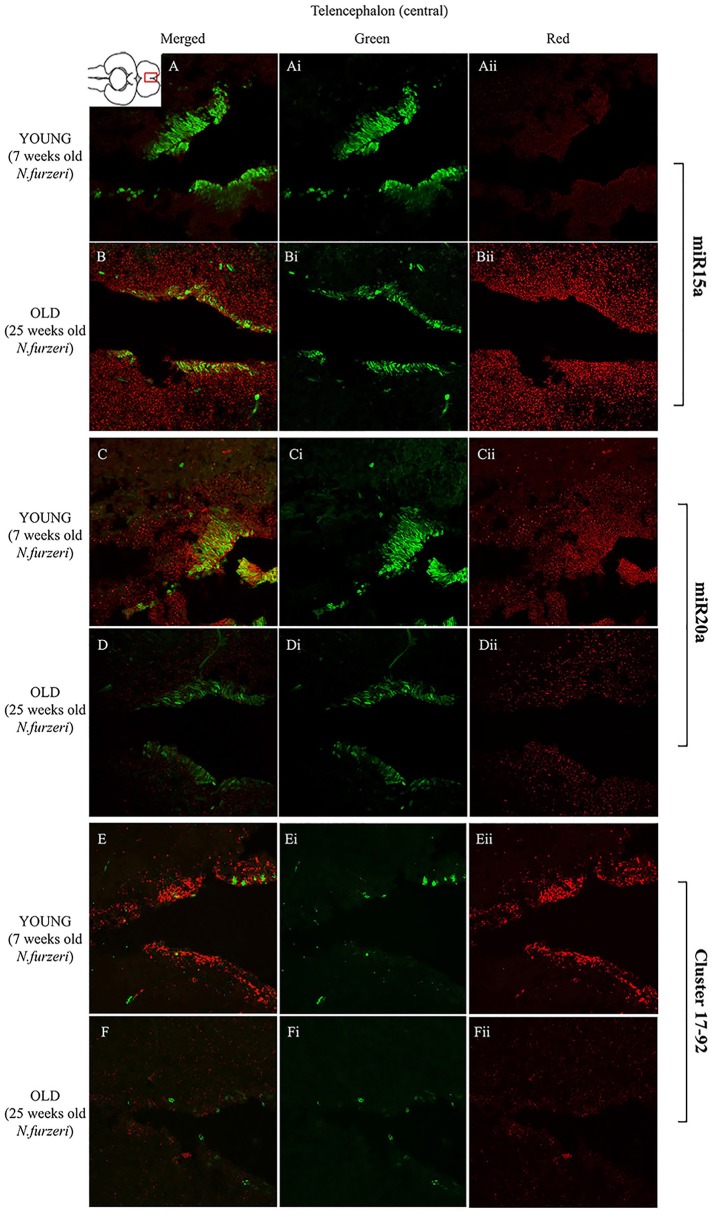
Magnification of the latero-posterior region of the telencephalon (lpTEL) stained in green for PCNA by IHC, and in red for miR15 (**A,B** strips), miR20 (**C,D** strips), and Cluster17–92 (**E,F** strips) by ISH. The lower inset on Aii shows an overview of a horizontal brain section: the location of the lpTEL is indicated by the red rectangle. The left column of the panel **(A–F)** shows the merged channels for the double staining; **A,B** refer to ISH for miR15a, respectively, in a young versus an old representative subject. **C,D** refer to ISH for miR20a, respectively, in a young versus an old representative subject. **E,F** refer to ISH for Cluster17–92, respectively, in a young versus an old representative subject. Central **(Ai–Fi)** and right **(Aii–Fii)** columns show the green and red single channel of the respective image on the left.

## Conflict of interest statement

The authors declare that the research was conducted in the absence of any commercial or financial relationships that could be construed as a potential conflict of interest.

